# The Geographical Distribution and Influencing Factors of COVID-19 in China

**DOI:** 10.3390/tropicalmed7030045

**Published:** 2022-03-06

**Authors:** Weiwei Li, Ping Zhang, Kaixu Zhao, Sidong Zhao

**Affiliations:** 1 Department of Landscape and Architectural Engineering, Guangxi Agricultural Vocational University, Nanning 530007, China; wwli2021@163.com; 2 College of Civil Engineering and Architecture, Jiaxing University, Jiaxing 314001, China; 3 College of Architecture and Urban Planning, Lanzhou Jiaotong University, Lanzhou 730070, China; 4 College of Urban and Environmental Science, Northwest University, Xi’an 710127, China; zhaokaixu@stumail.nwu.edu.cn; 5 School of Architecture, Southeast University, Nanjing 210096, China; 230189013@seu.edu.cn

**Keywords:** COVID-19, urban inequalities, infectious diseases, spatial distribution, China

## Abstract

The study of the spatial differentiation of COVID-19 in cities and its driving mechanism is helpful to reveal the spatial distribution pattern, transmission mechanism and diffusion model, and evolution mechanism of the epidemic and can lay the foundation for constructing the spatial dynamics model of the epidemic and provide theoretical basis for the policy design, spatial planning and implementation of epidemic prevention and control and social governance. Geodetector (Origin version, Beijing, China) is a great tool for analysis of spatial differentiation and its influencing factors, and it provides decision support for differentiated policy design and its implementation in executing the city-specific policies. Using factor detection and interaction analysis of Geodetector, 15 indicators of economic, social, ecological, and environmental dimensions were integrated, and 143 cities were selected for the empirical research in China. The research shows that, first of all, risks of both infection and death show positive spatial autocorrelation, but the geographical distribution of local spatial autocorrelation differs significantly between the two. Secondly, the inequalities in urban economic, social, and residential environments interact with COVID-19 spatial heterogeneity, with stronger explanatory power especially when multidimensional inequalities are superimposed. Thirdly, the spatial distribution and spread of COVID-19 are highly spatially heterogeneous and correlated due to the complex influence of multiple factors, with factors such as Area of Urban Construction Land, GDP, Industrial Smoke and Dust Emission, and Expenditure having the strongest influence, the factors such as Area of Green, Number of Hospital Beds and Parks, and Industrial NOx Emissions having unignorable influence, while the factors such as Number of Free Parks and Industrial Enterprises, Per-GDP, and Population Density play an indirect role mainly by means of interaction. Fourthly, the factor interaction effect from the infected person’s perspective mainly shows a nonlinear enhancement effect, that is, the joint influence of the two factors is greater than the sum of their direct influences; but from the perspective of the dead, it mainly shows a two-factor enhancement effect, that is, the joint influence of the two factors is greater than the maximum of their direct influences but less than their sum. Fifthly, some suggestions are put forward from the perspectives of building a healthy, resilient, safe, and smart city, providing valuable reference and decision basis for city governments to carry out differentiated policy design.

## 1. Introduction

### 1.1. Background

The scientific response to the global novel coronavirus pneumonia outbreak (hereinafter referred to as COVID-19) is a major scientific research task in the context of global public health events. It requires a close intersection of the subjects including medicine, geography, management, sociology and economics, and a rapid breakthrough in scientific theories and methods for dynamic surveillance, precise prevention and control, accurate prediction, and effective response to the epidemic outbreak. Major epidemic diseases directly threaten the economic and social development of all human beings. Summarizing and analyzing the spatial-temporal distribution patterns and regularities of infectious diseases [1], spread patterns [2,3], spatial effects [4,5], geographic impact and public health significance [6,7] are important research contents in geography and spatial epidemiology [8]. The cholera epidemic of 1832 in London gave impetus to the birth of modern urban planning, and the prevention and response to this major epidemic became an important origin for the development of urban planning [9,10]. Demographic, social, economic, infrastructural, and environmental conditions also vary across cities, and the COVID-19 epidemic has further exposed and even exacerbated such urban inequalities. Since spatial heterogeneity of infectious diseases often contains multiple opportunities to emerge from the crisis, it is necessary to find out the connections between COVID-19 spatial heterogeneity and urban inequality. Combating the epidemic crisis requires a comprehensive understanding of the interactions between multidimensional urban inequalities in macroeconomic, social, and environmental conditions and epidemics, and needs to reveal the complex driving mechanisms between COVID-19 spatial heterogeneity and urban inequality, which are crucial for policy design for sustainable urban development and public health. From the perspective of geography, planning and spatial epidemiology, the spatial spread of COVID-19 is a typical spatial evolutionary phenomenon and a process of interaction between people and nature, and it is of great theoretical value and practical significance to study the spatial differentiation pattern of COVID-19 and explore the comprehensive mechanism and strategy of epidemic control.

### 1.2. Literature Review

The development and evolution of infectious diseases are greatly influenced by the environment, so it is essential to figure out the spatial distribution characteristics and driving mechanism of COVID-19, the most serious infectious disease in recent 100 years [11]. Since the outbreak of COVID-19, many scholars from the studying fields of medicine, geography, management, sociology, and economics have been active in exploring this issue and produced a number of high-quality academic achievements, which have played a positive role in the judgment, decision making, treatment, and control of the epidemic. There are two shortcomings in the existing research results.

The first is that there is a lack of comprehensive studies with integration of economic, social, and environmental dimensions in spite of abundant research results. The main findings include that Neelon [12], Andersen [13] and Martines [14] analyzed the relationship between social vulnerability and COVID-19 incidence and death rates in the United States and Brazil; Paul analyzed the effects of black proportion, unemployment rate, residential segregation rate, rates of college or post-secondary education, HIV and diabetes incidence, and other social indicators on COVID-19 death rates in the U.S. [15]; Harlem examined the effects of overcrowding, low education level, fewer medical facilities, and more chronic diseases on COVID-19 infection rates in urban communities in New York [16]; Huang analyzed the effects of meteorological conditions and air quality on the association of COVID-19 infection and death rates in China [17]; Kan [18], Rojas [19], and Yip [20] analyzed the association between urban built environment and COVID-19 risk; Zeitoun analyzed the impact of care setting and social characteristics on COVID-19 death rates [21]. However, existing research mainly focuses on a single area of social or economic or built environment, while there are few comprehensive studies that integrate economic, social, ecological, and other factors into a unified framework. Only Luo [22] and Mollalo [23] have conducted exploratory studies based on an example of the United States.

The second is the single research method with insufficient diversity and influenced by covariance and methodological limitations, resulting in a small number of analysis factors and a very insufficient study of the interaction between factors. The existing research results are mainly based on regression analysis methods and linear models, including geographically weighted regression [24,25], Generalized Linear Model [26], spatial autoregressive model and conditional autoregressive model [27], Markovian Agents [28], correlation analysis and GIS mapping expression method [29], Geographically Weighted Principal Component Analysis [30], spatial autocorrelation [31,32], and Spatial Lag Model [33,34]. The intention to avoid the collinearity has led to a general lack of quantitative analysis indicators (5–8 independent variables) in existing research, which weakens the accuracy and application value of conclusions. More importantly, the spatial dynamics of COVID-19 consists of a variety factor, and the strength and even the nature of the effects of each factor are highly correlated with other factors, but most papers cannot quantitatively scale the factor interaction effects due to the limitations of the research methods.

### 1.3. Aim and Question

The existing research has shown that the unbalanced spatial distribution of COVID-19 is common, but its driving mechanism is still unknown. Different factors vary greatly in their influence on COVID-19 [35], and it is important to find the key factors and analyze the interaction between them, which is the foundation for constructing a spatial dynamics model of infectious disease spread and evolution, and the basis for developing scientific prevention and control strategies. This paper aims to investigate the following questions: ① How strong is the spatial heterogeneity of COVID-19? ② How and to what extent do various factors affect spatial differentiation of COVID-19 by integrating economic, social, and ecological environment dimensions? ③ How can the strength and nature of interaction between critical influencing factors be measured and further reveal or construct the driving mechanism of spatial differentiation of COVID-19? Geodetector is a powerful tool for analyzing spatial differentiation and its influencing factors, which has gotten attention from more scholars recently. Due to huge differences between cities in COVID-19 risk, health facilities and medical conditions, living environment, pollution and ecological construction, and economic development level, implementing a “one-size-fits-all” with ignorance of such differences is divorced from reality and will not lead to desirable results. A mastery of the geographical distribution and spatial differentiation characteristics of COVID-19 and its influencing factors is the basis for implementing city-specific policies, and Geodetector is an appropriate tool to conduct such research. To this end, this paper conducts an empirical study by means of Geodetector and tries to answer the above questions based on China study, with the aim to provide a theoretical basis and accumulating practical experience for scientific responses to COVID-19 and other similar international and regional infectious diseases such as Middle East Respiratory Syndrome (MERS), Severe Acute Respiratory Syndrome (SARS), Avian Influenza, Dengue, Ebola, and Cholera.

## 2. Research Design

### 2.1. Study Area: China

**Figure 1:**
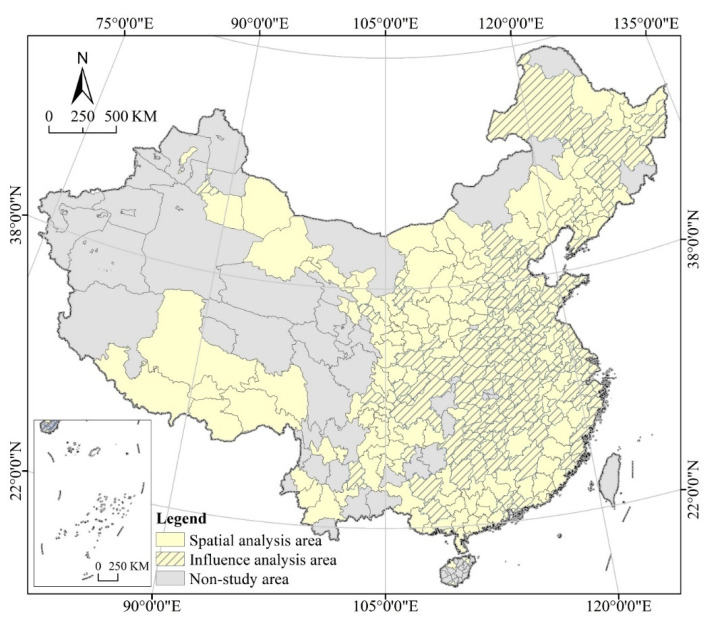
Study Area.

The study area includes 296 cities, excluding Sansha City, Hong Kong, Macao and Taiwan, as well as autonomous prefectures and county-level cities in the mainland (Figure 1). A total of about 103,000 patients are found in China, including about 85,000 in prefecture-level cities, about 5000 from overseas (with their cities not yet been determined), and the rest 13,000 in county-level or autonomous cities. As 29 March 2021, 281 cities reported infections and 71 reported deaths. We can identify the spatial distribution patterns and heterogeneity features of COVID-19 by spatial clustering, standard deviation ellipse, and spatial autocorrelation analysis tools of ARCGIS 10.2 (Geographic Information System, ESRI, USA). In a highly mobile society such as China, for cities with only one or a few infected cases, it is more likely that the infection is caused by inter-city mobility rather than by inter-transmission within the local population. To eliminate the influence of isolated points or random factors, this paper takes the median of 26 patients as the cutoff and selects 143 cities to carry out Geodetector research. Although Zunyi has more than 26 infected people, it was not included in the Geodetector analysis because of its incomplete collection of economic, social, and environmental statistics (independent variables).

The 143 sample cities are typically representative from the perspective of cities, infected people, the dead, and the world with 61 of them reporting deaths. From the perspective of cities, sample cities account for about 48% of all in the study area. From the perspective of infected people, sample cities account for about 98% in the study area. From the perspective of the dead, sample cities account for about 86% in the study area. From the perspective of the world, as of 29 March 2021, COVID-19 infections were reported in 214 countries and regions around the world, totally about 130 million, with an increase of more than 500,000 new confirmed cases per day, still in a rapid growth. China’s achievements in combating COVID-19 are leading in the world, and the Chinese experience is of great reference value for other countries in responding to COVID-19. The empirical study on the case of China is typical and representative.

### 2.2. Research Steps and Data Sources

**Figure 2:**
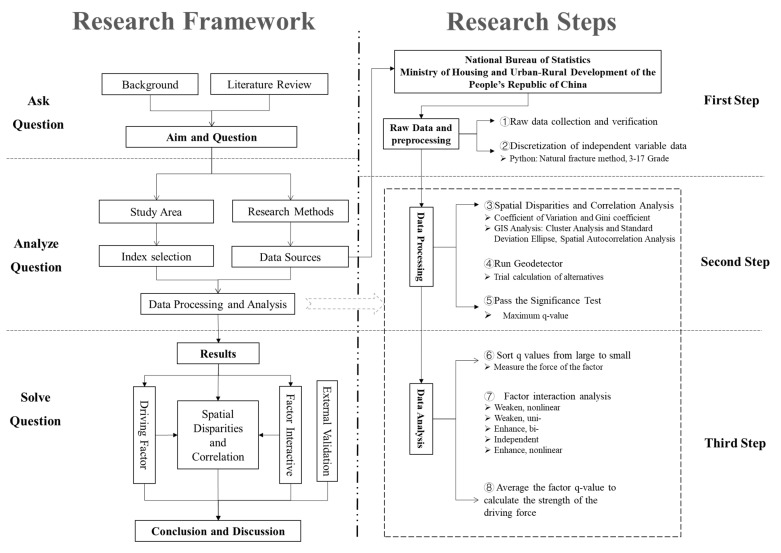
Research framework and steps.

Based on the research of relevant scholars, this study is mainly divided into three steps and eight key points (Figure 2) [36]. The first step is about Raw Data and preprocessing. ① Develop complete raw data tables based on the Baidu COVID-19 real-time big data report, urban economic, social, ecological and environmental statistics published on the websites of the China Statistics Bureau and the Ministry of Housing and Urban-Rural Development of the People’s Republic of China. The data mainly comes from the *China Urban Construction Statistical Yearbook* and the *China City Statistical Yearbook*, with some absent collected from statistical yearbooks, statistical bulletins, government work reports of provinces and cities. The COVID-19 data collected in this paper are as of 29 March 2020, while the others are as of 2019. ② Discrete processing must be performed before running Geodetector, due to the fact that the independent variable data are numeric. To eliminate the human influence, nature breaks are adopted in this paper. Discrete the continuous data of independent variables by Python (version 3.10.2 Python Software Foundation Beaverton, U.S.A.) and classify the 143 cities into 15 categories (3–17).

The COVID-19 epidemic is still not over, and virus mutation is ongoing, resulting in a number of new variants with greater transmissibility, pathogenicity, and high viral load such as Delta and Omicron. However, limited by many factors, this paper did not include them in the research framework for the following reasons: firstly, the research of this paper ended on 29 March 2020, while new high-risk variants such as Delta and Omicron were first discovered and reported in October 2020 and November 2021, respectively, not falling within the study time range; secondly, large bodies of city-scale economic, social and environmental data used in this paper are subject to a fixed cycle for collection and counting, and the latest data available have been adopted in the study; thirdly, new high-risk variants such as Delta and Omicron have aroused great vigilance in all countries, but their impact on China remains to be observed for a longer period of time, as China has effectively controlled the spread and dissemination of the new variant of the virus in the mainland China, thanks to the world’s strictest dynamic zero-case policy and the strict inspection system for all persons and substances entering China. In addition, experts such as Zhong Nanshan, Zhang Wenhong and Zhang Boli believe that China’s rapid response and dynamic zero-case policy can cope with various types of virus variants, and that as long as the whole society takes active measures to prevent and control the epidemic as required by the national and local governments, Delta and Omicron will not have a great impact on China, and the actual situation is largely consistent with the experts’ predictions.

The second step is about Data Processing. ③ Use Excel to calculate the coefficient of variation and Gini index of dependent variables and analyze the spatial differentiation level of COVID-19. ④ Import the raw data of the dependent variable and the discretized data of the independent variable into Geodetector software and calculate the analysis results. ⑤ Select the best alternatives in ②, and choose the one with the largest q-value as the final option under the condition of meeting the same or higher significance level. In this paper, we use 0.05 as the criterion for significance test and consider 0.1 in special cases, for exploratory studies under relaxed conditions.

The third step is about Data Analysis. Analyze and discuss the calculation results of ③ and ⑤. ⑥ Determine the strength of the explanatory power of the independent variables according to q-values. ⑦ Analyze the interaction effect between driving factors. ⑧ Average q-values for the independent variables that pass the significance test, calculate the strength of the 3 driving forces, and further reveal the driving mechanism of epidemic risk spatial distribution and its policy implications.

### 2.3. Research Methods and Index Selection

#### 2.3.1. Cluster Analysis and Standard Deviation Ellipse

Clustering is the process of dividing similar samples into different groups or more subsets by means of static classification or rule set, so that the member samples in the same subset all have similar properties. The standard deviation ellipse is one of the classical methods for analyzing the spatial distribution direction, with the size of the ellipse reflecting the concentration of all elements of the spatial pattern and the declination (semi-major axis) reflecting the dominant direction of the pattern. The elliptical semi-major axis indicates the direction of the data distribution, while the semi minor-axis indicates the range of data distribution. The data will have a more pronounced directionality when there is a larger difference (larger flattening) between the semi-major axis and the semi minor-axis. For the semi minor-axis of the standard deviation ellipse, its shorter length indicates a more concentrated geographical distribution of study objects and a stronger influence of agglomeration and centripetal forces on the spatial pattern [37,38]. This paper provides a visual analysis of the spatial pattern of geographic distribution of COVID-19 patients and deaths based on the cluster analysis and the standard deviation ellipse method of ARCGIS, thus presenting the spatial heterogeneity features of epidemic risk.

#### 2.3.2. Spatial Autocorrelation Analysis

The spatial autocorrelation index is an important index to measure the potential interconnectedness and dependence of study objects in a region. There are global spatial autocorrelation and local spatial autocorrelation. The former is to describe the spatial characteristics of the attribute values of spatial elements in the whole region and reflect the similarity of their neighboring attribute values, which is of great importance for the analysis and description of the distribution characteristics of a spatial attribute in the whole region; the latter is to calculate and analyze the correlation degree between a spatial object in the region and its neighboring objects, analyze the differences in the local characteristics of the distribution of study objects in space, and reflect the spatial heterogeneity and instability in the local region [39]. The common methods for spatial autocorrelation analysis include Moran’s I, Geary’s C, Getis, and Join count. In this paper, Moran’s I is used, consisting of Global and Local Moran’s I. The values of Global Moran’s I are in a range of [−1, 1]. At a given significance level (generally 5%), the value >0 indicates a positive spatial correlation, and a larger value indicates a more significant spatial correlation; the value <0 indicates a negative spatial correlation, and a smaller value indicates a greater spatial difference, while the value = 0 indicates a random spatial distribution. Local Moran’s I subdivide spatial correlation patterns into four types, specifically, H-H and L-L of a positive spatial correlation (that is, spatial units with high or below-average attribute values surrounded by domains with high or below-average attribute values), and H-L and L-H of a negative spatial correlation (that is, spatial units with high or below-average attribute values surrounded by domains with low or above-average attribute values) [40,41]. They are calculated based on the equation as follows:

(1) $$\mathrm{Global}\;\mathrm{Moran}’s\;I=\frac{N}{S_{0}}\times \frac{\sum_{i=1}^{N}\sum_{j=1}^{N}W_{ij}\left(Y_{i}-\bar{Y}\right)\left(Y_{j}-\bar{Y}\right)}{\sum_{i=1}^{N}{\left(Y_{i}-\bar{Y}\right)}^{2}},\;S_{0}=\sum_{i=1}^{N}\sum_{j=1}^{N}W_{ij}$$

(2) $$\mathrm{Local}\;\mathrm{Moran}’s\;I_{i}=\frac{Y_{i}-\bar{Y}}{\sum_{i=1}^{N}Y_{i}-{\bar{Y}}^{2}}\sum_{j=1}^{N}W_{ij}(Y_{i}-\bar{Y})$$

where, N represents the number of study objects, $Y_{i}$ and $Y_{j}$ are observed values of study objects i and j, $\bar{Y}$ is the average of $Y_{i}$, $W_{ij}$ is a spatial weight matrix, and $S_{0}$ is the sum of spatial weight. In this paper, ARCGIS 10.2 and GeoDa 1.18 are used for the spatial autocorrelation analysis, where the spatial weight matrix is a distance-based spatial weight matrix, and the parameters are all default values of the software. For the number of neighbors, the maximum value is 66, the minimum is 1, the mean is 32, and the median is 30. The distance is measured by the “Euclidean Distance”, and the specified bandwidth is 442,404 by the distance band method and the number of neighbors is 4 by the K-Nearest Neighbor method.

#### 2.3.3. Geodetector

In this paper we use the coefficient of variation and Gini coefficient for spatial heterogeneity measures and analyze the influence factors and their interaction effects based on the Geodetector, which was established by Professor Wang Jinfeng in 2010 as a new statistical method to detect spatial heterogeneity and reveal its influencing factors [42,43]. The geographically weighted regression is a linear model, while Geodetector is a nonlinear model. Geodetector can quantify the interaction force between two independent variables and two dependent variables without considering multicollinearity. At present, the method has been widely used in geography, sociology, economics, ecology, environment science, landscape science, planning science, and medicine as well as many other natural and humanities disciplines. In the study field of spatial differentiation of COVID-19 and its influencing factors, Wang [44], Wu [45], and Xie [46] conducted an exploratory study based on the Geodetector method.

The core idea of the Geodetector is based on the assumption that if an independent variable has a significant influence on a dependent variable, the spatial distribution of the independent and dependent variables should be similar. Geodetector offers 4 functions of factor detection, interaction detection, risk detection, and ecology detection (http://www.geodetector.cn (accessed on 21 April 2021)), and in this paper the first two functions are used to study the spatial differentiation of COVID-19 in China cities and its driving mechanisms. In other words, factor detection and interactive detection are conducted based on Geodetector with the populations diagnosed as COVID-19 infected and dead as the dependent variables $Y_{i}$, and the indicators related to economic, social, and ecological environment dimensions as the independent variables $X_{i}$. Factor detection enables the analysis of the influence of the independent variable $X_{i}$ on the dependent variable $Y_{i}$. The value of q, in a range of [0, 1], is used to represent the strength of influence, and a larger value implies a greater influence. With h representing the number of strata or classifications of the independent variables, $N_{h}$ and N representing the number of cities in stratum h and the study area, $\sigma_{h}^{2}$ and $\sigma^{2}$ representing the variance of the dependent variable in stratum h and the study area, respectively, SSW representing the Within Sum of Squares, and SST representing the Total Sum of Squares in the study area, the calculation equation for q is:

(3) $$q=1-\frac{\sum_{h=1}^{l}N_{h}\sigma_{h}^{2}}{N\sigma^{2}}=1-\frac{SSW}{SST},\;SSW=\sum_{h=1}^{l}N_{h}\sigma_{h}^{2},\;SST=N\sigma^{2}$$

The general way to identify the interaction is to add the product term of the two factors to the regression model and test its statistical significance. However, the two-factor interaction does not necessarily exhibit a multiplicative relationship. By calculating and comparing the q-values of single factors and the q-values of two superimposed factors separately, the Geodetector helps to determine whether there is an interaction between two factors, as well as the strength, direction, linearity, or nonlinearity of the interaction. The superposition of two factors involves multiplicative and other relationships and can be detected as long as there is a relationship. Interaction detection feeds back the interaction between two driving factors, that is, it detects whether the results are enhanced or weakened, or get independent of each other when $X_{i}$ and $X_{j}$ act. According to the strength of the interaction effect, the results are classified into 5 types [47] (see Table 1). “Non-linear Weaken” indicates that $X_{i}$ and $X_{j}$, when acting on Y at the same time, show mutual inhibition and are in an antagonistic state, and the combined influence of the two is less than that when they would be independent. “Unitary-non-linear Weaken” indicates that $X_{i}$ and $X_{j}$, when acting on Y at the same time, and the combined influence of the two is between the maximum and minimum values of independent influence. “Bifactor enhancement” indicates that $X_{i}$ and $X_{j}$, when acting on Y at the same time, their combined influence is greater than the maximum of their independent influences, but less than the sum of their independent influences. “Independent” indicates that $X_{i}$ and $X_{j}$, when acting on Y at the same time, the combined influence of the two factors is equal to the sum of their independent influence, that is, the influence of two independent variables on dependent variables is independent of each other. “Non-linear enhancement” indicates that $X_{i}$ and $X_{j}$, when acting on Y at the same time, the combined influence of the two factors is greater than the sum of their independent influences, implying there is a synergistic effect for the influence of the two independent variables on the dependent variable.

In the analysis of spatial heterogeneity of COVID-19, six indicators of number of patients, number of deaths, inspection rate per million population, death rate per million population, inspection rate per hundred square kilometers, and death rate per hundred square kilometers are used as observed variables. Based on the two indicators of the number of COVID-19 patients and the number of deaths, this paper explores the driving mechanism of spatial differentiation of COVID-19 with 15 indicators selected as independent variables from three driving forces of economy, society and ecological environment with reference to the research ideas of relevant scholars (see Table 2).

The spatial diffusion and distribution of COVID-19 is a complex process with many influencing factors and complicated relationships [48]. From the perspective of economic development, the high economic vitality in developed cities favors the spread and diffusion of the virus. Additionally, lagging economic development will affect the motivation of cities to control COVID-19, as well as the scale of investment in medical facilities and health resources [49]. In addition, the industrialization has a huge impact on its own development and that of the world for China as the “factory of the world”, and the surge in industrial orders in China after the outbreak of COVID-19 has put forward new requirements and challenges regarding the prevention and control of the epidemic for a large number of factories that have resumed work and production [50]. Therefore, Gross Domestic Product (GDP), Revenue, Expenditure, Per-GDP, and Number of Industrial Enterprises are adopted to represent the influence of the quantity and quality of urban economic development on the spread and distribution of the COVID-19 epidemic. Interpersonal interaction is crucial for urban residents, and higher population density will increase the chance of close human contact, leading to an increased likelihood of rapid “human-to-human” transmission of COVID-19; therefore, Population Density should be included in the research system [51]. COVID-19 is a typical major public health event characterized by suddenness, complexity, destructiveness and unpredictability, and its timely screening, effective control and scientific treatment are directly related to the life safety of urban residents, which poses a severe test to the emergency response capacity and carrying capacity of health and medical facilities in cities of different sizes [52]. Area of Urban Construction Land is a common indicator to measure the city size, while Number of Hospital Beds and Number of Licensed (Assistant) Doctors can effectively represent the carrying capacity of urban medical facilities, so it is necessary to include them in the research framework. As COVID-19 is mainly transmitted through air, the impact of urban air pollution and habitat settlement quality on COVID-19 should not be ignored. Industrial NOx Emissions and Industrial Smoke and Dust Emission are significant indicators for assessing pollution level, while Area of Green, Area of Parks, Number of Parks, and Number of Free Parks are key indicators to represent the quality of human settlement environment and ecological environment [53,54]. From the perspective of policy design, the value of scale control is higher than that of the ratio, so all indicators except per-GDP and Population Density are scale values.

## 3. Results

### 3.1. Spatial Heterogeneity and Relevance Analysis

**Figure 3:**
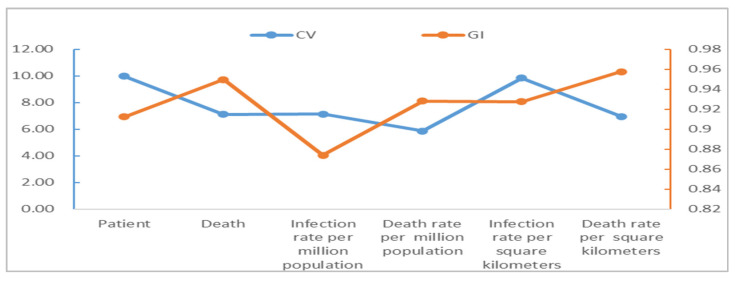
Spatial heterogeneity analysis of COVID-19 in China cities. Note: CV stands for coefficient of variation and GI stands for Gini index.

The spatial distribution of the populations diagnosed as COVID-19 infected and dead in China cities shows significant disparities, and they have basically maintained a good coordination (see Figure 3). Wuhan has the largest number of patients and deaths; Ningde, Kaifeng, Hanzhong and Ankang have the smallest number of patients, while 209 cities including Nanjing, Handan and Kaifeng have 0 death. The data used in this paper are from official statistics, and they have been reviewed by more than one department and are highly authoritative and accurate with little possibility that the deceased could be wrongly attributed. However, due to the presence of asymptomatic COVID-19 cases and the impossibility of conducting nucleic acid tests on all urban residents on a regular basis, there is a possibility that asymptomatic COVID-19 cases may be omitted from the data. The asymptomatic infections found account for a very low percentage of all infections at present, which is quite common for all cities, so their impact on the analysis results is negligible or tolerable. The coefficients of variation of patients are greater than 7, while the coefficient of variation of death is close to 10, all much greater than 0.36, reflecting high disparities [55,56]. The Gini index of patients is about 0.91 and that of death is up to 0.95, all much greater than 0.6 (according to the United Nations Development Programme, a value greater than 0.6 indicates a disparity), indicating a very uneven spatial distribution of COVID-19. Spatial heterogeneity in this paper is simply referring to differences in reported cases and COVID-attributable deaths between cities.

Spatial heterogeneity in patients and deaths is also significant from the prospective of population and patients per hundred square kilometers. From the prospective of patients per million population, Wuhan City had the largest number of patients and deaths, while Lincang City had the smallest number of patients and Zhoukou City had the smallest number of deaths. From the prospective of patients per hundred square kilometers, Wuhan City had the largest number of patients and deaths, while Yichun City had the smallest number of patients and Bayan Nur City had the smallest number of deaths. The coefficients of variation are 7.14 and 5.87 for the patients and deaths per million population, respectively, and 9.85 and 6.95 for the patients and deaths per hundred square kilometers, all significantly greater than 0.36. Their Gini index are 0.87, 0.93, 0.93, and 0.96, respectively, all greater than 0.6, indicating significant spatial differences in COVID-19 patients per million population and per hundred square kilometers. In addition, the comparison of coefficient of variation and Gini index shows that the infection rate and death rate per hundred square kilometers have the strongest spatial heterogeneity, while the infection rate and death rate per million population have the weakest, with the numbers of patients and deaths lying in the middle.

**Figure 4:**
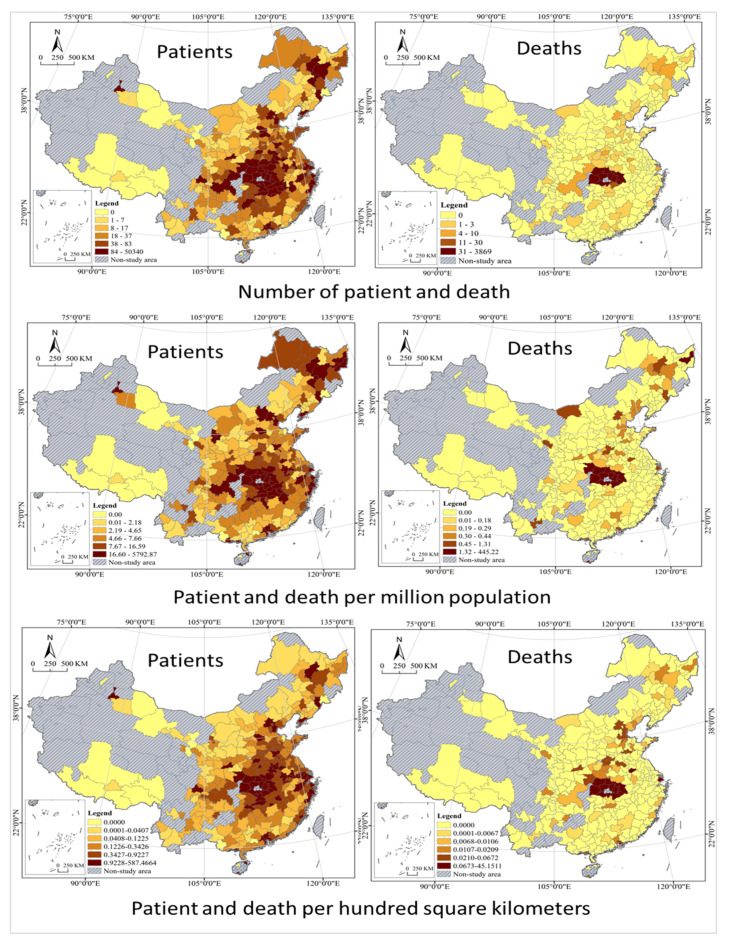
Cluster analysis of COVID-19 in China cities.

Based on the GIS quantile spatial cluster analysis, after excluding the 0 value, the study area is classified into five types, that is, Higher, High, Medium, Low, and Lower (see Table 3 and Figure 4). Quantile classification does not have empty classes, and all values are arranged from small to large and divided into multiple classes in statistical analysis of data, each containing the same number of samples. Accidents are divided into four categories according to the *Regulations on the Reporting*, *Investigation and Disposition of Work Safety Accidents*, and those involved are held responsible according to the type of accident. By the Regulations, an accident that has caused the deaths of less than 3 persons is an ordinary accident; an accident that has caused the deaths of at least 3 but less than 10 persons is a large accident; an accident that has caused the deaths of at least 10 but less than 30 persons is a serious accident; and an accident that has caused the deaths of at least 30 persons is an especially serious accident. With reference to the classification idea above, cluster analysis was conducted on the geographical distribution of COVID-19 deaths with thresholds of 3, 10, and 30. The analysis shows that patients with significant disparities in geographic distribution, and there are two centers formed. The south is a large center with a circle structure, while the northeast is a small center, which is not well developed, only a rudiment with an axial belt structure. Most of the Higher cities in the south are located in Hubei Province, with a few including its western neighbor Chongqing and those in Shaanxi Province, which are concentrated and contiguous in distribution. With Higher as the center of a circle, High cities are distributed in the south, north and east of it, showing strong spatial agglomeration. Medium, Low and Lower cities are distributed in a graded manner at the periphery, with Higher cities as the center in general; while High, Medium, Low and Lower cities are distributed like ripples, showing the characteristics of cascade and circle. Higher cities in the northeast are in Heilongjiang province and expand to cities southward in Jilin Province and northwestward in Inner Mongolia. The geographical distribution of Deaths presents very high disparities; however, the spatial pattern is different from that of patients. It only forms a center in the south, with Higher cities all located in Hubei Province, High cities as its western neighbors, Medium and Low cities scattered and randomly distributed. It is important to note that most of the Lower cities are those with zero death, and their spatially concentrated contiguous distribution is the result of geographical factors and has little to do with the epidemic itself. It should be noted that the patients per million population and per hundred square kilometers are basically the same as all infected groups in terms of spatial differentiation characteristics, with only local differences in some cities, such as cities in the northeast, Bohai rim, and southwest regions. However, the death per million population and per hundred square kilometers differ significantly from total deaths in spatial differentiation patterns, for example, the northeastern cities have a higher death rate per million population, while Beijing-Tianjin-Hebei urban agglomerations have a higher death rate per hundred square kilometers.

**Figure 5:**
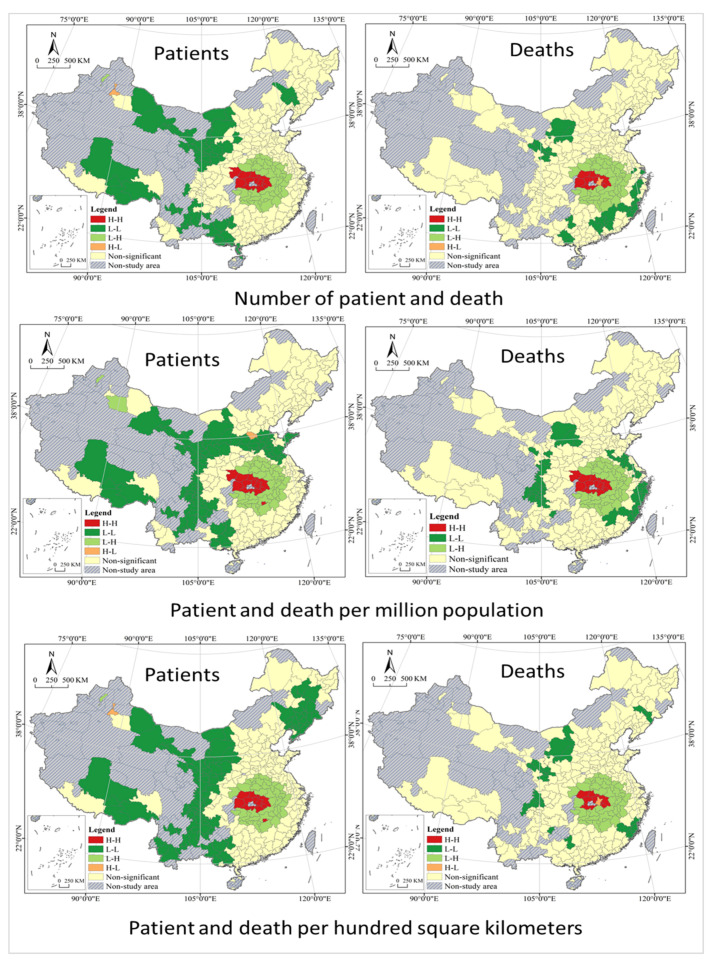
Spatial autocorrelation analysis of COVID-19 in China cities.

In terms of spatial correlation, the global spatial autocorrelation analysis shows a positive spatial correlation and dependence of COVID-19 risks in China cities, and the local spatial autocorrelation analysis shows a large difference in their regional distribution (see Figure 5). All values of Global Moran’s I are greater than zero at 5% or a more stringent level of significance, and a smaller value indicates a positive spatial distribution with a lower spatial correlation. The Global Moran’s I index implies that there is indeed some spatial correlation between the spread and distribution of the COVID-19 epidemic in China, but it is not significant in general, and there is no spatial pattern of circular distribution centered on a highly infected city. The phenomenon may be the result of strict quarantine restrictions, specifically, under China’s adherence to a dynamic zero-case policy, the city will restrict its contact with others to varying degrees in case of any infections found there, thus blocking the spatial spread of the virus. Local Moran’s I is used to analyze the geographical distribution characteristics in COVID-19 risk cities of four types of H-H, H-L, L-L, and L-H, further revealing the spatial heterogeneity and instability of China cities in different regions. In terms of infection risk, the spatial heterogeneity pattern of H-H, L-H, H-L stays stable in general, but L-L shows a large variation and difference in the northeast. In terms of death risk, the spatial patterns of H-H and L-H are generally stable, but L-L shows a large variation in coastal and southwestern cities, and there is no H-L type of deaths per million population.

**Figure 6:**
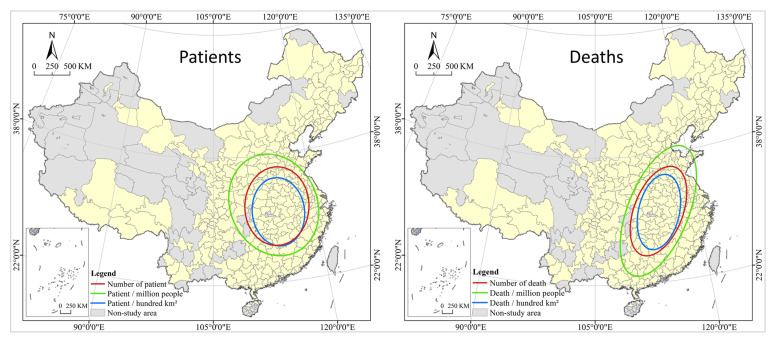
Standard deviation ellipse analysis of COVID-19 in China cities.

The results of the local spatial autocorrelation analysis show that COVID-19 has a high spatial correlation and spillover effect in local regions of China. A city with large numbers of infections and deaths is also at great risk for the spread of the virus in their surrounding areas for the following two reasons: firstly, COVID-19 is a virus transmitted through close contact, and according to the first law of geography, cities that are closer to each other have a higher degree of connection and interaction, leading to the virus imperceptibly spreading with people and logistics in the economic and social cycle between central cities and surrounding cities; secondly, since the implementation of the new urbanization strategy, the central and local governments have issued a series of policies to support urbanization in nearby areas, forming a circulation in population movement dominated by short-distance trips with provincial capitals and core cities at the center, which facilitates the spread and diffusion of the virus in a small region. In addition, the spatial distribution of patient is mainly north-south and northwest-southeast, and the death is mainly northeast-southwest, and the direction of the latter is more prominent than the former (see Figure 6).

### 3.2. Influencing Factors and Effect Analysis

Geodetector is a great tool for measuring, mining, and analyzing spatial heterogeneity of COVID-19 geographic distribution. The functional modules of “factor detector” and “interaction detector” in Geodetector software enable the quantitative test of the consistency of spatial distribution pattern between independent variables (15 indicators) and dependent variables (COVID-19 infections and deaths). Geodetector has stronger power than general statistics, which is based on correlation, but has a stronger hint of causality. Since it is much more difficult to keep the geospatial distribution of the independent and dependent variables consistent than to keep the consistency of their mathematical modeling curves, in this paper we use the results of the Geodetector analysis to represent the influence of the 15 independent variables on the geographic distribution of COVID-19, with the “factor detector” results used to reveal the direct influence and the “interaction detector” results used to find the synergistic influence.

Direct influence reveals that the geographical transmission and spread of COVID-19 infections and deaths are affected by multiple factors, and there is a large difference in the influencing factors and their influence between the two (Table 4). The results of “factor detector” show that 12 and 8 factors in the 15 independent variables have different influences on the spatial distribution pattern of COVID-19 infections and deaths. The mean values of the influence factors are 0.35 and 0.20, with maximum values of 0.50 and 0.32, and minimum values of 0.19 and 0.10, respectively. For infections, economic factors have the greatest influence, while ecological and environmental factors have the least, with social factors in the middle. For deaths, economic, ecological, and environmental factors are equally influential, and more influential than social factors. With reference to the mean and extreme values (maximum and minimum) of factor influence, the influencing factors can be classified into three types of critical, significant, and accessory factors, as detailed below:

For the infections, GDP, Area of Urban Construction Land, Expenditure, and Industrial Smoke and Dust Emission have great influence, with q-values around 0.5, much higher than those of other factors, and can be considered as critical influencing factors, indicating that city size, economic strength, government investment, and air pollution control are the primary factors that must be included in decision-making and analysis for “city-specific” response to and control of COVID-19. Number of Hospital Beds, Industrial NOx Emissions, Area of Green, and Number of Parks have moderate influence, with q-values around 0.3, close to the mean, and can be considered as significant influencing factors, indicating that improving urban medical facilities, making the human living environment better and building green spaces deserve the attention of policy designers. Number of Industrial Enterprises, Number of Licensed (Assistant) Doctors, Area of Parks, and Number of Free Parks are less influential, with q-values around 0.2, well below the mean, and can be considered as accessory influencing factors. The assumption that Per-GDP, Population Density, and Revenue are consistent with the geographic distribution of COVID-19 is not statistically significant, and their direct influence can be neglected.

For the deaths, the critical influencing factor is Expenditure only, unlike those for the infections, and there is a significantly reduced number of factors. GDP, Number of Hospital Beds, Industrial Smoke and Dust Emission, Area of Green are significant influencing factors, Revenue, Area of Urban Construction Land, Industrial NOx Emissions are accessory influencing factors; Per-GDP, Number of Industrial Enterprises, Population Density, Number of Licensed (Assistant) Doctors, Area of Parks, Number of Parks, and Number of Free Parks have negligible influence. The number of significant and accessory influencing factors is close in the deaths compared to the infections, but their components and forces change considerably.

**Figure 7:**
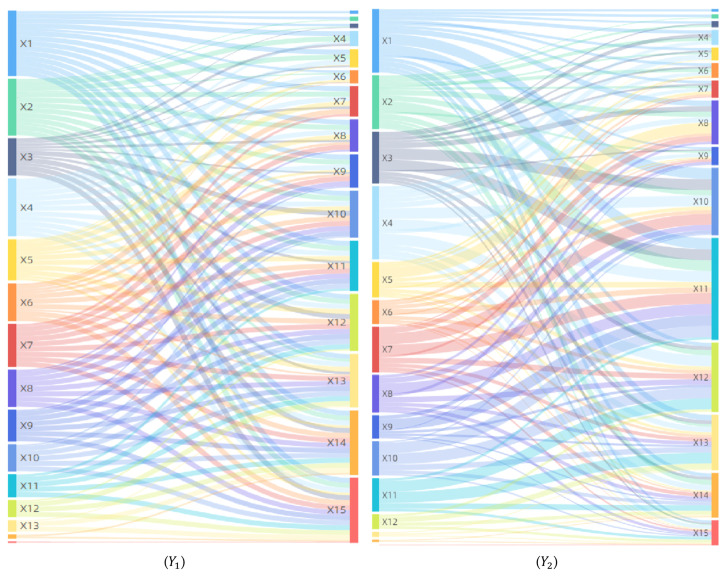
Analysis of interaction detector. (***Y*_1_**), (***Y*_2_**): Number of Patients and Deaths.

In terms of synergistic influence, factors are in a complex interaction relationship and have a comprehensive influence on the geographic spread and diffusion of COVID-19 (Figure 7). The interaction detector has shown that the pairwise interactions between factors are non-linear enhancement and bifactor enhancement, and there is no independent or attenuated relationship, indicating that the economic, social, ecological, and environmental factors have complex and intricate driving effects on the spatial distribution and evolution of COVID-19. For patients, including 93 non-linear enhancement factor pairs and 12 bifactor enhancement factor pairs. The non-linear enhancement effect is dominant, indicating that when the two factors jointly affect COVID-19, the interactive influence is greater than the sum of their direct influences. Out of non-linear enhancement factors, the high impact factor pairs with interaction forces greater than 0.98 account for about 90%, such as $X_{1}$∩$X_{7}$, $X_{1}$∩$X_{15}$ and $X_{4}$∩$X_{5}$.For deaths, including 45 non-linear enhancement factor pairs and 60 bifactor enhancement factor pairs. The bifactor enhancement effect is dominant, indicating that when two factors jointly affect COVID-19, the interactive influence is greater than the maximum of their direct influences but less than their sum. Both per capita GDP ($X_{2}$) and population density ($X_{6}$) have negligible direct influence on infections and deaths, but they have a great influence on the geographical distribution and spatial differentiation of COVID-19 through their interaction with other factors, especially the interactive influences of the factor pairs $X_{2}$∩$X_{4}$, $X_{2}$∩$X_{5}$, $X_{6}$∩$X_{7}$, and $X_{6}$∩$X_{11}$ are greater than 0.98, leading to both being factors that cannot be neglected in the development and implementation of epidemic prevention policies.

## 4. Discussion

### 4.1. High Spatial Heterogeneity of COVID-19 in the Spatial Distribution and Spread

Spatial stratified disparities, known as the first law of geography and one of the three basic principles of ecology, is used to analyze geographic phenomena where the variance within spatial layers is smaller than that between layers. This study argues that the spatial distribution of COVID-19 in China’s cities is highly heterogeneous, that is, COVID-19 varies significantly between different cities in China. It is highly concentrated in certain cities, with a high level of “polarization”, and a significant “Matthew effect”, which is in agreement with the conclusion of Cheng [57]. It may be quite common as Sartorius [58], Manley [59], Ahmed [60], and Alexander [61] have found that the geographic distribution of COVID-19 cases and deaths in England and the United States is also highly heterogeneous, and Beare [62] finds in the study that the geographic distribution of COVID-19 in the United States conforms to a power law with a Pareto effect, showing a highly uneven geographic distribution of COVID-19 in the United States. Furthermore, we have found that the distribution of COVID-19 in China cities is spatially autocorrelated, which is agreement with Saffary’s view that there is a similar spatial correlation in U.S. county-level cities [63]. Overall, the spatial spread and evolution of COVID-19 is not random. Therefore, it is of great practical value to know and characterize the spatial differentiation of economic, social, ecological, and environmental factors and their effects on COVID-19 for preventing and controlling the spatial spread and evolution of COVID-19.

### 4.2. High Complexity of COVID-19 Spatial Differentiation Driving Factors and Their Interaction Effects

**Figure 8:**
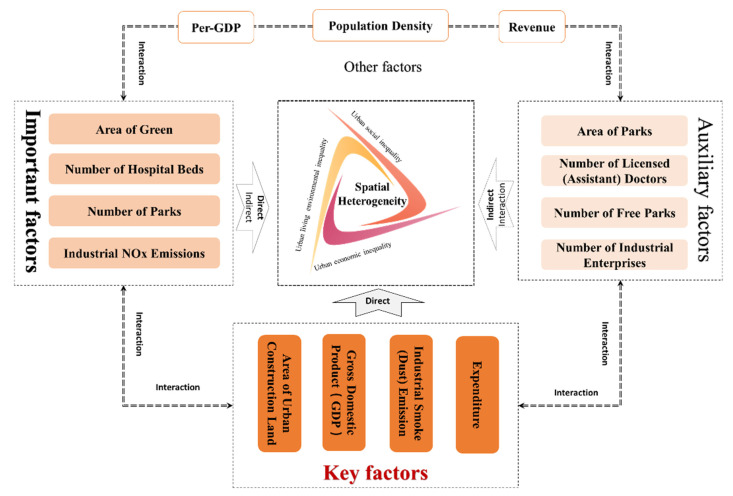
Driving mechanism of spatial heterogeneity.

There are many driving factors affecting the spatial differentiation of COVID-19, with significant difference in strength and strong interaction between each other, showing a prominent trend of diversification of COVID-19 spatial spread and evolution drivers and complexity of driving mechanisms. Based on the ranking of driving forces of spatial differentiation of COVID-19 patients and death, and the mean value of the forces (0.35), the driving factors are divided into four categories, and then the driving mechanism of spatial differentiation of COVID-19 in China cities is constructed (see Figure 8) with the interaction effects between the factors taken into account. The first category is Key factors, that is, TOP4 factors. These have the greatest impact on the spatial differentiation of COVID-19, with the direct driving forces all higher than the average. Such factors include Area of Urban Construction Land, Gross Domestic Product (GDP), Industrial Smoke and Dust Emission, and Expenditure. The second category is Important factors. Their driving forces are lower than average but all higher than 0.3. They have a non-negligible direct impact on the spatial differentiation of COVID-19 with an indirect impact beginning to take on. Such factors include Area of Green, Number of Hospital Beds, Number of Parks, and Industrial NOx Emissions. The third category is Auxiliary factors. They have a weak direct effect, while mainly playing an indirect and interactive role. Such factors include Area of Parks, Number of Licensed (Assistant) Doctors, Number of Free Parks, and Number of Industrial Enterprises. The fourth category is other factors. Their direct effect can be ignored, and they mainly play an indirect role by relying on the interaction with other factors. Such factors include Per-GDP, Revenue, and Population Density.

Some of the findings in this paper corroborate some viewpoints of existing papers. Previous studies have shown that there are large spatial differences in the distribution of COVID-19, and that urban medical conditions, greening and living environment quality, pollution, economic development, and other factors have an undeniable influence on the spread of the virus. This study further confirms these views. As of 29 March 2020, 214 and 199 countries (regions) across the world reported COVID-19 infections and deaths, respectively, with the coefficient of variation at the national scale of 4.20 and 3.82, and the Gini index of 0.87 and 0.88, respectively, suggesting that there is great variation in the global spread and distribution of COVID-19 virus. At the same time, Mizumoto [64] believes that there is a statistical correlation between health facilities and COVID-19 infection rate based on multiple regression analysis; Xie [65] holds that the quantity of hospital beds and medical workers has a positive impact on the spatial differentiation of COVID-19; Deguen [66], Pei [67], Ramirez [68], Kazakos et al. [69] conclude that environmental pollution and meteorological conditions such as PM2.5, NOx and air temperature are in a significant correlation with the spatial distribution of COVID-19.

However, some results are different or even opposite, and these new findings are of great value to complement and refine the spatial spread driving mechanism and evolution law of COVID-19. The differences are mainly in three areas: first of all, for the economic dimension, Amdaoud [70], Paez [71], and Lin [72] argue that GDP per capita is highly correlated with COVID-19 death rate in EU countries, which is different from the finding in this paper. The finding shows that GDP per capita plays a significant driving role for spatial heterogeneity among COVID-19 patients under loose condition, but there is no such relationship among deaths. Secondly, from the social dimension, Sarria-Guzman [73], Baser [74], and Fortaleza et al. [75] hold that the correlation between population density and the spread of the COVID-19 is above the significance level, which is different from the finding in this paper. The finding shows that although population density has a strong interaction with other factors, its direct driving effect has not passed the significance test. Thirdly, from the ecological and environmental dimensions, Liu [76] argues that environmental factors have no significant impact on the spread of COVID-19, which is contrary to the finding in this paper. The finding shows that environmental factors, especially Industrial Smoke and Dust Emission are key factors. As put by Thomas [77] and Miranda [78], the inconsistency of findings may be due to the scale effects or the sensitivity of research methods. Geodetector is sensitive to geographic scale and spatial stratification, and differences in scale and stratification may lead to significant changes in local regions or domains. Besides, unlike other countries around the world, China has adopted diverse and strict quarantine policies in various cities to control the spread of COVID-19. Some studies show that these quarantine policies do have good results, but due to the limitation of data acquisition, this paper does not include them in the research framework [79]. The results of this study are different from those of other countries, which may be due to the absence of quarantine policy analysis. In summary, there are numerous driving factors of COVID-19 spatial heterogeneity, and there are interaction effects of bifactor enhancement and nonlinear enhancement between factors, reflecting the high complexity and scaling property of the driving mechanism.

### 4.3. Precise Policy Design Oriented to Core Drivers and Their Interaction Effects

Epidemic prevention policy and medical spatial planning are the most critical policy tools for the government to control the spatial spread and dissemination of COVID-19, and the core work of management, planning, geography, and spatial epidemiology. The driving factors of COVID-19 spatial propagation and evolution are quantitatively large, and the factor forces and interactions are significantly heterogeneous, complex, and dynamic. Therefore, this paper suggests a precise, refined, and differentiated policy design for spatial governance of COVID-19 based on the driving mechanism constructed in the previous sections with reference to the findings of Peleg [80] and Grzybowski et al. [81], to make a shift in policy design and implementation from “one-size-fits-all” and “extensive” to “differentiated” and “precise”.

The first is to, in line with the ecological civilization construction requirements, increase investment in green space and park construction, make more parks free to citizens, and improve the positive shelter capabilities of public spaces such as schools, gymnasiums, exhibition halls, auditoriums, organize regular exercises and drills to strengthen the emergency isolation and storage capacity of public spaces, and enhance the comprehensive emergency response capabilities for transforming public spaces to treatment and nursing space [82,83]. Further suggested policy would be to promote industrial transformation and upgrading and energy conservation and green development, strengthen the control of industrial pollution, strictly limit the total emission of Industrial Smoke, Dust and NOx, and reduce the risk of COVID-19 being transmitted or exacerbating the patient’s condition by air pollutants based on the findings of Yao [84], Wang [85], Yu [86], and Man [87].

The second is to implement the development concept of healthy and resilient cities, increase financial investment in medical space and optimize the spatial allocation of medical facilities according to supply characteristics and requirements, provide more training and supply for medical workers, expand medical staff and increase hospital beds, and enhance the emergency resilience of medical space and facilities; and implement the concept of smart development, control the size of cities in accordance with the requirements of “tightening up increment, revitalizing the stock and improving the quality”, and strictly control the expansion and spreading of urban construction land to achieve efficient spatial governance based on the findings of Li [88], Zhou [89] and Wang [90].

The third is to implement the development concept of safe cities and smart cities, promote the application of smart technology in urban construction, prepare and implement the specialized plan for urban prevention and control of infectious diseases, establish technical standards for epidemic prevention, treatment, and emergency response, and build a safety control system of “source control + tailing margin” for cities [91,92]. The urban space margin was retained in the past mainly to serve urban economic development and ecological construction, used for uncertain major industrial and strategic projects and special ecological or livelihood projects. In the context of the COVID-19 epidemic, it is important to integrate development and safety in the future, to promote urban space retention in favor of risk control and safety. The city should strengthen the safety margin of infectious disease prevention and control facilities and treatment space and provide sufficient space to support the addition of specific medical institutions and facilities or the establishment of shelter hospitals and temporary emergency isolation sites.

## 5. Conclusions

Affected by the scale, there is an urgent need to strengthen multi-scale empirical research to develop a precise and scientific epidemic prevention policy for different age groups, social classes, exposure levels, and different realities such as “city closure”, “community closure”, “strict control of overseas import”, “resumption of production and schooling”, based on different scales of geographic units such as countries, regions, cities, counties, towns and communities, and special spaces, and the potentially large differences in the level of spatial heterogeneity of COVID-19 spread and evolution and its driving mechanisms. This paper investigates the spatial heterogeneity of COVID-19 at the urban scale and its driving mechanism based on quantitative analysis and finds that the spatial distribution and spread of COVID-19 are highly spatially heterogeneous, with significant differences in the driving forces and their interactions. It further classifies the driving factors into three categories, stating that Area of Urban Construction Land, Gross Domestic Product (GDP), Industrial Smoke and Dust Emission, Expenditure are the most influential factors; the effect of Area of Green, Number of Hospital Beds, Number of Parks, Industrial NOx Emissions cannot be ignored; Area of Parks, Number of Licensed (Assistant) Doctors, Per-GDP, Population Density play indirect roles mainly by means of interaction.

This study not only provides useful reference for China and other countries in the world, such as the United States, India, Brazil, France, the United Kingdom, Italy, and Turkey with more severe outbreaks, to actively explore the COVID-19 spatial distribution patterns, transmission mechanisms and dispersal models, evolutionary dynamics and mechanisms, prevention and control recommendations and countermeasures, and new approaches to spatial governance and policy design, but also offers new ideas and frameworks for the study of Middle East Respiratory Syndrome (MERS), Severe Acute Respiratory Syndrome (SARS), Avian Influenza, Dengue, Ebola, Cholera and other international and regional infectious diseases. The empirical studies based on the ideas and framework provided in this paper can reveal the spatial spread patterns and evolution laws of these infectious diseases and their driving mechanisms, which is invaluable for Middle East and African countries to formulate scientific prevention and control policies, especially with a great value of application for less developed countries such as Syria, Iraq, Afghanistan, Iran, Yemen, Libya, Sudan, South Africa, Ethiopia, Nigeria, and Angola, which are suffering from war or virus ravages.

There are significant differences in COVID-19 risk and response performance among different cities and countries around the world, as well as factors influencing the spread and geographic distribution of the virus and its prevention and control strategies. The research framework and ideas constructed in this paper can be applied to the analysis of cities in the United States, India, Brazil, Iran and other countries to explain the spatial spread and geographical distribution patterns of COVID-19 and provide a basis for the design and implementation of infectious disease control policies. In addition, there are obvious inequalities between different countries in the dimensions of economic development stage, medical and health conditions, living environment and pollution, which have different impacts on the spatial transmission and distribution of COVID-19 epidemic. With multi-scale empirical studies on different countries, regions, and cities, as well as comparative analysis of multiple cases across the world by multi-methods, GIS and network analysis methods [93], this paper enables explaining and predicting patterns of infectious diseases across geographical space and identifying areas of potentially high risk, to help better allocate limited health services and resources and further enrich spatial epidemiological research.

There are also some deficiencies in our study, which may have a certain impact on the accuracy and applicability of the conclusions. Due to the limitation of data acquisition, comparative studies at different scales and in different time periods are not covered in this paper, and differences in epidemic prevention policies and population attributes between cities are not included in the research framework. In the era of “post-epidemic” or “new epidemic” or even “new-new epidemic”, we call for more international scholars to carry out cross-national multi-scale and multi-time series comparative and empirical studies to get more precise conclusions, to enable policy makers, planners, and the public to fully know the spatial distribution of COVID-19 and its driving mechanisms, and provide a theoretical basis and practical guidance to conquer the virus earlier and create a better life.

## Figures and Tables

**Table 1:** Interaction between Explanatory Variables.

Graphical Representation	Description	Interaction
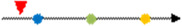	$q\;(X_{i}\cap X_{j})\;<\;\mathrm{Min}\;(q\;(X_{i}),\;q\;(X_{j}$))	non-linear Weaken
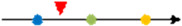	$\mathrm{Min}\;(q\;(X_{i}),\;q\;(X_{j}))\;<\;q\;(X_{i}\cap X_{j})\;<\;\mathrm{Max}\;(q\;(X_{i})),\;q\;(X_{j}$))	unitary-non-linear Weaken
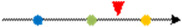	$q\;(X_{i}\cap X_{j})\;>\;\mathrm{Max}\;(q\;(X_{i}),\;q\;(X_{j}$))	bifactor enhancement
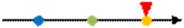	$q\;(X_{i}\cap X_{j})=q\;(X_{i})+q\;(X_{j}$)	Independent
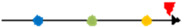	$q\;(X_{i}\cap X_{j})\;>\;q\;(X_{i})+q\;(X_{j}$)	non-linear enhancement

Legend: 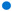 Min (q ($X_{i}$), q ($X_{j})$); 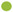 Max (q ($X_{i}$), q ($X_{j}$)); 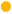
q ($X_{i}$) + q ($X_{j}$); 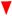 q ($X_{i}$∩$X_{j}$).

**Table 2:** Model variable description.

Variable	Index	Code	Type
Dependent variable $Y_{i}$	Number of Patients	$Y_{1}$	
	Number of Deaths	$Y_{2}$	
Independent variable $X_{i}$	Gross Domestic Product (GDP)	$X_{1}$	Economic driving force
	Per-GDP	$X_{2}$	
	Revenue	$X_{3}$	
	Expenditure	$X_{4}$	
	Number of Industrial Enterprises	$X_{5}$	
	Population Density	$X_{6}$	Social driving force
	Area of Urban Construction Land	$X_{7}$	
	Number of Hospital Beds	$X_{8}$	
	Number of Licensed (Assistant) Doctors	$X_{9}$	
	Industrial NOx Emissions	$X_{10}$	Ecological and Environmental driving force
	Industrial Smoke and Dust Emission	$X_{11}$	
	Area of Green	$X_{12}$	
	Area of Parks	$X_{13}$	
	Number of Parks	$X_{14}$	
	Number of Free Parks	$X_{15}$	

**Table 3:** The Value of Global Moran’s I.

		Global Moran’s I	Confidence Level
Number of patient and death	Patients	0.009	0.008
	Deaths	0.002	0.010
Proportion of patient and death per million population	Patients	0.030	0.004
	Deaths	0.011	0.009
Proportion of patient and death per hundred square kilometers	Patients	0.010	0.013
	Deaths	0.003	0.023

**Table 4:** Analysis of factor detector.

			$Y_{1}$		$Y_{2}$	
			q-Statistic	Influence	q-Statistic	Influence
economic	Gross Domestic Product (GDP)	*X*_1_	0.49 **	0.39	0.18 **	0.20
	Per-GDP	*X*_2_	0.21		0.08	
	Revenue	*X*_3_	0.06		0.11 **	
	Expenditure	*X*_4_	0.49 **		0.32 **	
	Number of Industrial Enterprises	*X*_5_	0.19 **		0.05	
social	Population Density	*X*_6_	0.09	0.36	0.02	0.17
	Area of Urban Construction Land	*X*_7_	0.50 **		0.10 **	
	Number of Hospital Beds	*X*_8_	0.33 **		0.24 **	
	Number of Licensed (Assistant) Doctors	*X*_9_	0.25 **		0.07	
ecological and environmental	Industrial NOx Emissions	*X*_10_	0.32 **	0.32	0.13 **	0.20
	Industrial Smoke and Dust Emission	$X_{11}$	0.49 **		0.24 **	
	Area of Green	$X_{12}$	0.33 **		0.24 **	
	Area of Parks	$X_{13}$	0.26 **		0.11	
	Number of Parks	$X_{14}$	0.32 **		0.07	
	Number of Free Parks	$X_{15}$	0.19 **		0.00	

Note: ** stands for *p* < 0.05.

## Data Availability

The data used in this paper mainly come from the China City Statistical Yearbook, China Statistical Yearbook published national bureau of statistics and China Urban Construction Statistical Yearbook issued by the Ministry of Housing and Urban-Rural Development of the People’s Republic of China. Most of the data can be obtained by visiting the following links: http://www.mohurd.gov.cn/xytj/tjzljsxytjgb/jstjnj/ and http://www.stats.gov.cn/tjsj/ndsj/ (access on 5 March 2021). The COVID data of each city comes from Baidu, which can be obtained by visiting the following links: https://voice.baidu.com/act/newpneumonia/newpneumonia (access on 29 March 2021).
